# Effect of Autoclave Cycles on Surface Characteristics of S-File Evaluated by Scanning Electron Microscopy

**DOI:** 10.7508/iej.2016.01.006

**Published:** 2015-12-24

**Authors:** Hamid Razavian, Pedram Iranmanesh, Hamid Mojtahedi, Rahman Nazeri

**Affiliations:** a *Department of Endodontics, Torabinejad Dental Material Research Center, Dental School, Isfahan University of Medical Sciences, Isfahan, Iran; *; b * General Dentist, Isfahan University of Medical Sciences, Isfahan, Iran; *; c * Student Research Committee, Dental School, Isfahan University of Medical Sciences, Isfahan, Iran*

**Keywords:** Autoclave, Endodontic Instruments, Root Canal Therapy, Scanning Electron Microscopy, Surface Characteristic

## Abstract

**Introduction:**

Presence of surface defects in endodontic instruments can lead to unwanted complications such as instrument fracture and incomplete preparation of the canal. The current study was conducted to evaluate the effect of autoclave cycles on surface characteristics of S-File by scanning electron microscopy (SEM).

**Methods and Materials:**

In this experimental study, 17 brand new S-Files (#30) were used. The surface characteristics of the files were examined in four steps (without autoclave, 1 autoclave cycle, 5 autoclave cycles and 10 autoclave cycles) by SEM under 200× and 1000× magnifications. Data were analyzed using the SPSS software and the paired sample t-test, independent sample t-test and multifactorial repeated measures ANOVA. The level of significance was set at 0.05.

**Results:**

New files had debris and pitting on their surfaces. When the autoclave cycles were increased, the mean of surface roughness also increased at both magnifications (*P*<0.05). Moreover, under 1000× magnification the multifactorial repeated measures ANOVA showed more surface roughness (*P*<0.001).

**Conclusion:**

Sterilization by autoclave increased the surface roughness of the files and this had was directly related to the number of autoclave cycles.

## Introduction

The aim of root canal treatment by mechanical instruments is to create a clean disinfected canal [1]. The S-File is a NiTi hand file with cylindrical core design similar to H-File; however, it has double cutting edges and thicker shaft than H-File, which minimizes the possibility of fracture [2]. Some studies have shown that manual instruments have higher capability for canal cleaning than engine-driven files [3, 4]. Ahlquist *et al.* [4] compared the performance of S-File and ProFile and reported that S-file was able to leave less debris in the apical region of the canal. 

Infection control processes are essential parts of modern dentistry. Since endodontic instruments are in contact with blood and mucosa, they need to be sterilized before use [5]. Sterilization should be carried out to remove all pathogenic organisms [6]. Using autoclave is a common technique for sterilization of all files which is able to demolish all bacteria, spores and viruses [7]. 

However, due to thermal expansion and contraction processes, sterilization in autoclave can permanently deform the flutes and make all cutting tools dull [8]. These surface changes can affect the properties of the file and result in unfavorable fracture of the instrument during root canal treatment [9]. The surface changes of the file include flattened flutes, piled up grooves, deformed tip and corrosion. Should these changes occur, the file must be discarded [10]. Research has shown that surface cracks and corrosion of the file can result in the instrument fracture [11-13]; hence the surface quality of the file is important. 

Scanning electron microscopy (SEM) is used for extensive structure analysis through secondary electron imaging [14] and is an excellent technique for analysis of surface characteristics [9]. Martins *et al.* [15] showed that the majority of the defects of endodontic files can be detected by SEM at high magnification. 

Lack of knowledge about the surface characteristics of the file can lead to complications such as file fracture and fault in canal preparation. Therefore, it is necessary to perform studies on the surface changes of the files. The present research was an attempt to investigate the effect of autoclave cycles on surface characteristics of S-File by means of SEM.

**Figure 1:**
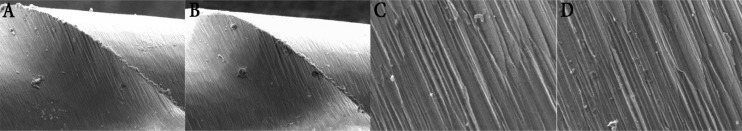
Surface characteristics of S-File; *A)* before autoclave cycle (200×); *B)* after 10 autoclave cycles (200×); *C)* before autoclave cycle (1000×) and *D)* after 10 autoclave cycles (1000×)

## Materials and Methods

In this experimental study, 17 new as-received #30 S-Files (Sendoline Co, Worecester, United Kingdom) with no crack and fracture were selected through simple sampling. Each file was investigated in four stages: without autoclave (control), and after 1, 5 and 10 autoclave cycles. 

To make similar conditions for all files, a class B autoclave (206 H, Farazmehr, Isfahan, Iran) was used with temperature of 121^°^C and 15 psi pressure for 20 min. The files were placed in the autoclave packs for sterilization with a tag indicating the completion of the sterilization process. After the completion of sterilization, the files were left to dry in a petri dish at room temperature for 45 min. 

In order to replicate similar imaging area in all stages, a scratch was made on the end of each file shaft by a diamond bur (835, TizKavan, Qazvin, Iran) installed on high speed handpiece (NSK, Nakanishi Inc, Japan). The surface roughness of the files was evaluated at 3 mm from the tip of the file by DS-130 electron microscopy (International Scientific Instruments, Inc., Pleasanton, CA, USA) under 200× and 1000× magnifications before and after sterilization (Figures 1A to D). 

**Figure 2:**
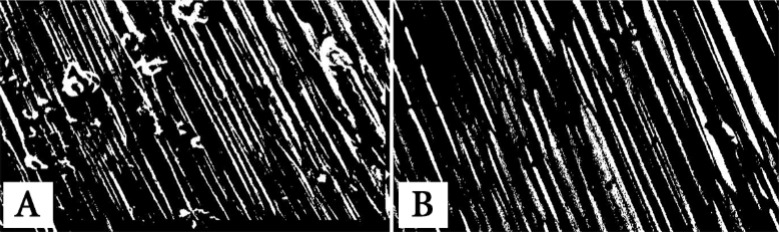
*A)* high and *B)* low thresholding SEM image

Mathworks Matlab R2013b software (New Mexico University, New Mexico, USA) was used for the analysis of images. To quantify the surface characteristics of each file, segmentation techniques were utilized for image processing. First, the brightness uniformity algorithms were used to uniform the brightness intensity of different images. In addition, the working distance of each image was measured as a reference distance and then the image was resized in accordance with its reference distance. The surface characteristics of the files were obtained by improved images. To this end, by applying high image thresholding the bumps were shown as bright areas with high pixel values (Figure 2A), and by applying low image thresholding, the grooves were detected as bright areas with high pixel values (Figure 2B). Finally, sum of pixels (indicating grooves and bumps) were used as the criteria for quantitative analysis of surface characteristics of the files. 

The obtained data were analyzed with SPSS software (SPSS version 20.0, SPSS, Chicago, IL, USA) and evaluated with the paired sample t-test, independent sample t-test and multifactorial repeated measures ANOVA. The level of significance was set at 0.05.

## Results

Prior to sterilization by autoclave, the files had debris and roughness at both magnifications. Further the multifactorial repeated measures ANOVA showed that by increasing the number of autoclave cycles, the mean of surface roughness of the files increased, indicating a significant difference at both magnifications (*P*<0.001). 

The results of paired sample t-test for intra-group analysis showed no significant difference for 1 and 5 autoclave cycles at 200× magnification (*P*=0.057). However, a significant difference was reported for all other stages (*P*<0.05). According to the independent t-test, the mean of surface roughness of the files was significantly higher at 1000× magnification compared to 200× magnification in all stages (*P*<0.05) (Table 1).

## Discussion

Previous studies have mostly focused on the effect of autoclave on mechanical properties, resistance to fracture and cutting power of endodontic instruments [16-19]. However, it is necessary to examine the surface quality of the files owing to their effect on the

instrument resistance to corrosion and fracture. Based on the novelty of S-File and favorable results of previous a study about this file [4], the present study evaluated the surface characteristics of S-File before and after sterilization by autoclave. 

**Table 1.** Mean (SD) of instrument surface roughness in total and at separate magnifications

	**Magnification**		**Surface roughness**	***P*** **-value**
	**200× **	**1000× **		
**0 autoclave cycles**	2.36 (1.29)^a^	21.66 (9.43)^d^	12.01 (11.87)	0.001
**1 autoclave cycles**	3.84 (2.70)^b^	32.66 (10.64)^e^	18.25 (16.60)	0.007
**5 autoclave cycles**	7.82 (8.38)^b^	53.83 (17.27)^f^	30.83 (27.05)	0.050
**10 autoclave cycles**	562.53 (362.30)^c^	5117.84 (2733.66)^g^	2840.18 (3010.43)	0.002
***P-*** **value†**	0.001	0.001	0.001	

a Multifactorial repeated measures of ANOVA test (comparison of surface roughness of files at each magnification);

b Independent sample t-test (paired comparison of surface roughness of files at two magnifications); Identical letters indicate insignificant difference

In the study conducted by Van Eldic *et al.* [20], it was shown that the file size had no effect on sterilization. Further, Moradi *et al.* [21] reported no difference in the microleakage of canals prepared up to files #20, 25 and 30. Hence, in the present study only file #30 was used, which is usually used as the master apical file in root canal treatments [22, 23].

Koehler and Hefferren [7] indicated that sterilization of root canal instruments with adequate heat is an appropriate method for extensive destruction of bacteria with minimum damage to the instrument. Furthermore, Stach *et al.* [24] showed that autoclave is the best instrument for sterilization of the tools with minimum corrosion rate. Therefore, in the current study the effect of using autoclave on surface characteristics of the files was analyzed. 

The findings of this study showed that new files have debris and roughness before sterilization by autoclave, which is similar to the results reported by Alexandrou *et al.* [25] and Spangnuolo *et al*. [26]. This may be due to the errors of production and/or packaging in the company. Thus, clinicians need to carefully check the surface defects of the files even the new as-received ones to prevent the consequent complications. 

The results of present research indicated that surface roughness increased after sterilization in autoclave, and this roughness was correlated with the number of autoclave cycles. Alexandrou *et al.* [25] investigated the effect of dry sterilization on surface characteristics of rotary files and reported a rise in surface roughness with an increase in the number of sterilization cycles. Also, Spagnuolo *et al.* [26] examined the effect of autoclave cycles on surface changes of rotary instruments and concluded that autoclave cycles changed the surface topography of the file and increased the surface roughness with an increase in autoclave cycles. In another study, Valois *et al.* [9] evaluated the impact of autoclave sterilization cycles on surface characteristics of nickel-titanium rotary files. They reported that autoclave cycles enhanced the depth of surface roughness. Although these studies have evaluated the rotary files, their findings are indicative of the similar effect of sterilization cycles on surface characteristics of the files. Generally, the sterilization process can lead to surface roughness, deepened cavities and microscopic cracks in the tools, which consequently result in lower tolerance of the instruments against stresses and fracture. 

The results of the current study indicated the significant increase of surface roughness in files after 5 autoclave cycles, which is in line with the findings of Valois *et al.* [9]. These results were obtained under laboratory conditions; however, surface changes in files may occur with fewer autoclave sterilization cycles in clinical settings. So, frequent use of file and numerous autoclave cycles (over 5 cycles) are not suggested. 

Given the proven ability of SEM in detecting surface changes [15], in the present study surface characteristics of the files were analyzed under two magnifications. The diagnostic ability to detect changes was higher at 1000× magnification in comparison with 200× magnification in all cases. This indicates the importance of magnification in detecting the defects: the higher the magnification is, the more accurate the records of surface changes will be.

Due to high cost of SEM, the effect of autoclave on surface characteristics of one type of files was examined in the present study. Therefore, similar studies are recommended to be performed on other files. Moreover, similar studies are necessary to be conducted on files in clinical conditions.

## Conclusion

New files had debris and roughness before sterilization. The surface roughness significantly increased with an increase in the number of autoclave cycles. The surface roughness significantly increased after 5 autoclave cycles, so the files should be discarded after five autoclave cycles. Scanning electron microscopic images with higher magnification detected more roughness.
